# Inhibition of Aurora-B suppresses osteosarcoma cell migration and invasion

**DOI:** 10.3892/etm.2014.1491

**Published:** 2014-01-20

**Authors:** XIAO PING ZHU, ZHI LI LIU, AI FEN PENG, YUN FEI ZHOU, XIN HUA LONG, QING FENG LUO, SHAN HU HUANG, YONG SHU

**Affiliations:** 1 Department of Anesthesia, First Affiliated Hospital of Nanchang University, Nanchang, Jiangxi 330006, P.R. China; 2 Department of Orthopedics, First Affiliated Hospital of Nanchang University, Nanchang, Jiangxi 330006, P.R. China; 3 Jiangxi University of Traditional Chinese Medicine, Nanchang, Jiangxi 330004, P.R. China; 4 Department of Pathology, Cancer Hospital of Jiangxi Province, Nanchang, Jiangxi 330029, P.R. China

**Keywords:** osteosarcoma, Aurora-B, metastasis, AZD1152

## Abstract

Previous studies have suggested that Aurora-B may be involved in cancer metastasis. However, its role has been poorly evaluated in osteosarcoma (OS). The aim of this study was to investigate the correlation between Aurora-B expression and metastasis in human OS. The human OS cell line, U2-OS, and OS biopsy specimens were used in the study. The expression of Aurora-B protein was examined using immunohistochemistry and western blotting in OS tissues and U2-OS cells, respectively. AZD1152-hydroxyquinazoline-pyrazol-anilide, an inhibitor of Aurora-B, was used to inhibit Aurora-B expression in U2-OS cells. The effect of Aurora-B inhibition on U2-OS cell proliferation, invasion and migration was assessed using MTT, colony formation, wound healing and Transwell assays. The results showed that positive expression of the Aurora-B protein was observed in the nucleus, and that Aurora-B expression levels in the cases with pulmonary metastases were significantly higher than in those without metastasis. *In vitro*, the proliferation, invasion and migration of U2-OS cells were suppressed by the inhibition of Aurora-B. These results suggest that Aurora-B may be involved in OS metastasis, and may be a promising target in the treatment of OS metastasis.

## Introduction

Osteosarcoma (OS) is the most common tumor in bone and the third most common tumor in childhood and adolescence. Following the identification of effective chemotherapeutic agents, the five-year survival rates for patients treated with intensive multidrug chemotherapy and aggressive local control have been reported to be 55–80% (1–3). However, an improvement in survival rates has been limited only to patients with a high grade of disease. Patients with metastatic disease have a poor prognosis, particularly those with pulmonary metastases at diagnosis, with various studies reporting five-year survival rates of only 17–23% (4,5). Therefore, it is necessary to determine the mechanisms contributing to the metastasis of OS.

Aurora-B is an important protein kinase and is involved in the efficient execution and fidelity of mitosis. As part of the chromosomal passenger complex (CPC), Aurora-B has been shown to be involved in a number of mitotic functions, including chromosome-microtubule interactions, sister chromatid cohesion, the spindle-assembly checkpoint and cytokinesis. Previous studies have shown that Aurora-B is upregulated in several types of human cancer, and that upregulation correlates with poor prognosis. As a result, Aurora-B has been suggested to be an important antitumor target (6–9). Li *et al* (10) showed that downregulation of Aurora-B is capable of inhibiting proliferation and metastasis, inducing G2/M phase arrest in clear cell renal cell carcinoma cells and exerting antitumor activity in an SN12C xenograft model (10). In addition, a number of studies have indicated that nuclear Aurora-B expression is markedly associated with and involved in tumor metastasis (11–14). However, whether Aurora-B is involved in OS metastasis has yet to be elucidated.

In the present study, the expression of Aurora-B in OS with and without pulmonary metastasis was evaluated using immunohistochemistry (IHC). Furthermore, the effect of Aurora-B inhibition on cell proliferation, invasion and migration *in vitro* was investigated. AZD1152-hydroxyquinazoline-pyrazol-anilide (HQPA) was used to inhibit Aurora-B expression in U2-OS cells. Cell proliferation, migration and invasion were investigated using MTT, colony formation, wound healing and Transwell assays. The results revealed that there was a positive correlation between Aurora-B expression in OS tissues and pulmonary metastasis, and that cell proliferation, invasion and migration were inhibited by the inhibition of Aurora-B. The results indicated that Aurora-B may be involved in OS metastasis.

## Materials and methods

### Patients and specimens

A total of 60 samples were obtained from patients with OS of the extremities who underwent surgery in The First Affiliated Hospital of Nanchang University (Nanchang, China). The examination of pulmonary metastasis was performed using plain films and chest computed tomography (CT) scans at initial diagnosis. None of the patients had a history of previous therapies with antitumor drugs or radiotherapy. There were 14 cases with pulmonary metastasis (23.3%), while 76.7% of cases were without metastasis. The samples were fixed with 10% formalin, embedded in paraffin and then cut into 4-μm sections. Informed consent was obtained from all participants, and the study protocol was approved by the Institutional Ethics Committee (Jiangxi, China).

### IHC

Histological sections (4-μm) were stained with hematoxylin and eosin (H&E) and examined using IHC. IHC was performed using a streptavidin-peroxidase procedure. Briefly, antigen retrieval was performed by heating the deparaffinized, rehydrated sections in 10 mM citrate buffer (pH 6.0) for 20 min, followed by blocking with 10% goat serum. The sections were then incubated overnight at 4°C with the primary antibody (rabbit anti-Aurora-B monoclonal antibody; Abcam, Cambridge, UK) at a final dilution of 1:500. For the negative controls, the sections were incubated with phosphate-buffered saline (PBS) instead of antibodies. After being washed three times with PBS, the sections were incubated with biotinylated secondary antibody for 40 min and then incubated with horseradish peroxidase (HRP)-conjugated streptavidin for 30 min. The sections were subsequently subjected to chemiluminescent staining and counterstained using hematoxylin. The stained sections were evaluated and scored by two doctors of pathology, in a blind manner and without prior knowledge of the clinical pathological features of the patients. The expression levels of Aurora-B were judged according to staining intensity, following the examination of ≥500 cells in five representative areas, and the intensity scores were recorded as follows: None, 0; weak, 1; moderate, 2; and intense, 3. According to the percentage of tumor cells that were positive for Aurora-B expression, the following percentage scores were assigned: 0% (score 0); >10% (score 1), 11–50% (score 2), 51–80% (score 3), and 81–100% (score 4). The final score was averaged with the scores from the two doctors of pathology; these scores were calculated by adding the intensity score to the percentage score. A final score of <4 was defined as (−), while scores of 4 and 5 were defined as (+) and (++), respectively, and a score of ≥6 was defined as (+++).

### Cell lines and cell culture

The U2-OS human OS cell line was obtained from the American Type Culture Collection (Manassas, VA, USA), and the cells were routinely cultured in Dulbecco’s modified Eagle’s medium (DMEM; HyClone™, Thermo Fisher Scientific, Inc., Waltham, MA, USA) supplemented with 10% fetal bovine serum (FBS; Sigma, St. Louis, MO, USA) in a humidified 37°C incubator containing 5% CO_2_.

### Cell growth assay

U2-OS cells were cultured in 96-well tissue culture plates at a cell density of 5,000 cells per well in Minimum Essential Media (MEM; Invitrogen Life Technologies, Carlsbad, CA, USA) containing 10% FBS and 2 mM L-glutamine. Following attachment overnight, the medium was replaced and the cells were incubated with increasing concentrations (0, 5, 10, 50, 100 and 500 nM) of AZD1152-HQPA for 24, 48 and 72 h. Subsequently, MTT assays were performed in triplicate at a wavelength of 490 nm.

### Colony formation assay

U2-OS cells (1×10^6^/ml/well) were seeded in tissue culture plastic dishes and treated with AZD1152-HQPA (100 nM) for two weeks to form colonies. The formed colonies were stained with Giemsa, and the colonies containing >50 cells were counted under an inverted microscope (TE2000; Nikon, Tokyo, Japan). Six independent experiments were performed over multiple days.

### Western blot analysis

U2-OS cells in the exponential growth phase were treated with AZD1152-HQPA at various concentrations (0, 10, 50 and 100 nM) for 24 h. Total protein from the cells was extracted using radioimmunoprecipitation assay lysis buffer containing 60 μg/ml phenylmethylsulfonyl fluoride. Protein concentrations were assessed using a bicinchoninic acid protein assay kit (Boster Biological Technology, Ltd., Wuhan, China). The protein samples were denatured at 100°C for 10 min and then preserved at −20°C for later use. The proteins were separated by SDS-PAGE and transblotted onto polyvinylidene difluoride membranes. The membranes were then probed with rabbit anti-Aurora-B monoclonal antibody (1:500; Abcam) or β-actin antibody (1:2,000; Cell Signaling Technology Inc., Danvers, MA, USA) overnight at 4°C. Following incubation with the appropriate anti-rabbit or anti-mouse HRP-conjugated secondary antibody (1:5,000; Boster Biological Technology, Ltd.) for 1.5 h at room temperature, immunoreactive bands were visualized using chemiluminescence dissolvent (Thermo Fisher Scientific, Inc.) and exposed to X-ray film (Kodak, Rochester, NY, USA). The assessment of the grayscale values was performed using ImageJ (National Institutes of Health, Bethesda, MD, USA). All experiments were repeated six times over multiple days.

### Transwell assay

The invasion of U2-OS cells was measured using the BD BioCoat™ BD Matrigel™ Invasion Chamber (BD Biosciences, Franklin Lakes, NJ, USA) in accordance with the manufacturer’s instructions. The medium in the lower chamber contained 5% FBS as a source of chemoattractants. Cells were suspended in serum-free medium containing 100 nM AZD1152-HQPA and added to the upper chambers at the same time. The cultures were rinsed with PBS and the medium was replaced with fresh medium alone or medium supplemented with 10% FBS. The cells were then incubated at 37°C for 24 h. Cells that passed through the Matrigel-coated membrane were stained with Diff-Quik (Sysmex Corp., Kobe, Japan) and photographed. Cell migration was quantified using direct microscopic visualization and counting. The values for invasion were obtained by counting three fields per membrane and represented the average of six independent experiments performed over multiple days.

### Wound healing assay

Cell migration was assessed by examining the ability of the cells to move into a cellular space in a two-dimensional *in vitro* ‘wound healing assay’. In brief, cells were grown to confluence in six-well tissue culture plastic dishes to a density of ~5×10^6^ cells/well. Following treatment with 100 nM AZD1152-HQPA for 24 h, the cells were denuded by dragging a rubber policeman (Fisher Scientific, Hampton, NH, USA) through the center of the plate. Cultures were rinsed with PBS and the medium was replaced with fresh medium alone or medium containing 10% FBS. The cells were then incubated at 37°C for 24 h. Photographs were taken at 0 and 24 h, and the migration distance was measured. The cell migration rate was obtained by counting three fields per area and represented the average of six independent experiments performed over multiple days.

### Statistical analysis

All measurement data are presented as the mean ± standard deviation. Statistical analysis was performed using the independent-samples t-test, and the two-independent-samples test was used for the analysis of the correlation between Aurora-B protein expression levels and pulmonary metastasis. P<0.05 was considered to indicate a statistically significant difference. All analyses were performed using SPSS statistical software version 13.0 (SPSS, Inc., Chicago, IL, USA).

## Results

### Correlation between Aurora-B protein expression levels in OS tissues and pulmonary metastasis

**Figure 1:**
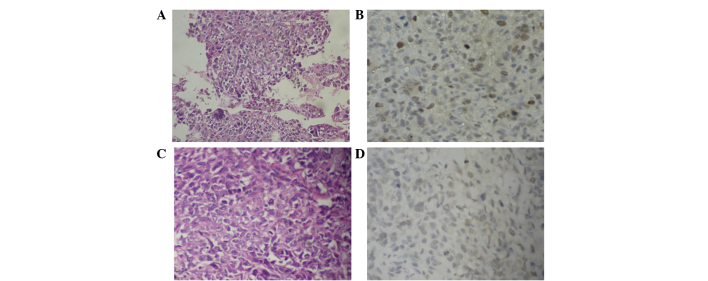
Aurora-B protein expression in OS with and without pulmonary metastasis (magnification, ×400). Representative images of (A) H&E staining for OS tissues with pulmonary metastasis, showing that OS is cell rich and has significant cellular atypia, anisonucleosis, prominent nucleoli and an abundant cytoplasm; (B) IHC staining for Aurora-B protein with lung metastasis, showing brown-yellow particles deposited in the nucleus and coloring of the majority of the cells; (C) H&E staining for OS tissues without pulmonary metastasis, showing that OS is cell-rich and has significant cellular atypia, anisonucleosis, prominent nucleoli, an abundant cytoplasm and a small quantity of bone-like matrix; (D) IHC staining for Aurora-B protein in OS tissues without pulmonary metastasis, showing brown-yellow particle deposition in the nucleus and coloring of only a few cells. OS, osteosarcoma; H&E, hematoxylin and eosin; IHC, immunohistochemistry.

Aurora-B was expressed in the nucleus (Fig. 1), and the positive expression rate was 53.3%. Notably, the positive expression rate of Aurora-B protein in the cases with pulmonary metastasis was 78.6.% (11/14), which was significantly different from that of the cases without pulmonary metastasis 45.7% (21/46). This indicated that Aurora-B may be involved in OS metastasis.

### Effect of Aurora-B inhibition on U2-OS cell proliferation in vitro

**Figure 2:**
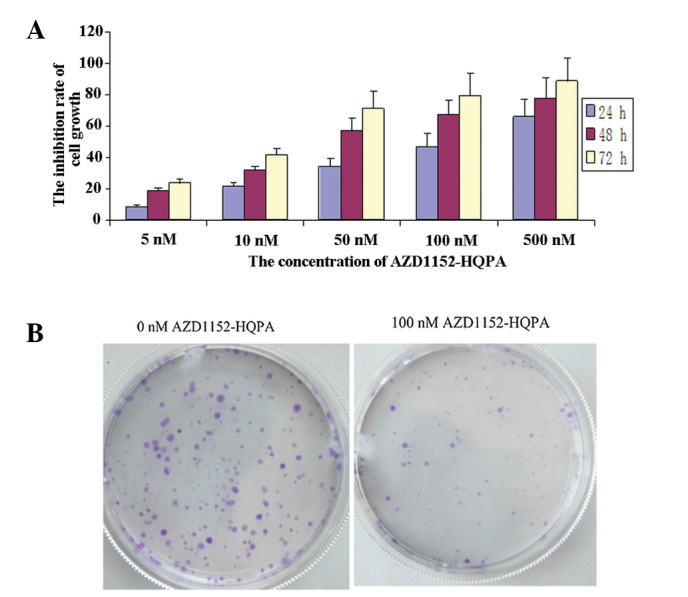
Inhibitory effect of AZD1152-HQPA on the growth of U2-OS cells. (A) MTT assays showed that AZD1152-HQPA inhibited U2-OS cell growth in a time- and dose-dependent manner. (B) Representative images of colony formation assays, showing that the colony formation rate in cells treated with 100 nM AZD1152-HQPA was lower than that in untreated cells. OS, osteosarcoma; HQPA, hydroxyquinazoline-pyrazol-anilide.

**Figure 3:**
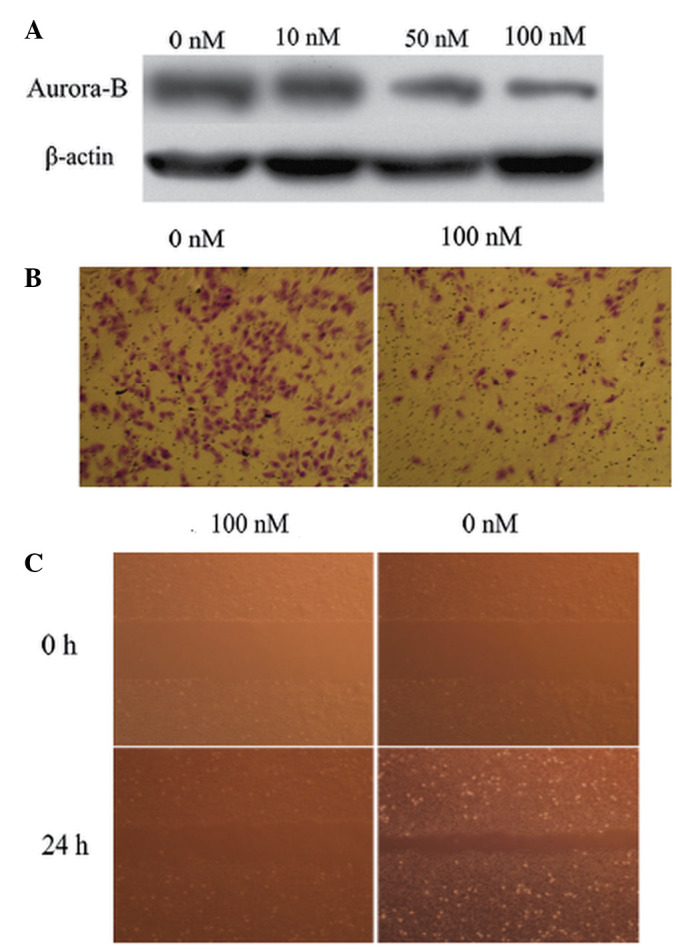
Effect of Aurora-B inhibition on U2-OS cell invasion and migration *in vitro*. (A) Representative images of western blot analysis of Aurora-B protein expression in U2-OS cells. Aurora-B protein expression in U2-OS cells was inhibited by AZD1152-HQPA in a dose-dependent manner. (B) Representative images of Transwell invasion assays, showing that invasion of U2-OS cells was inhibited by Aurora-B inhibition. (C) Representative images of the wound healing assay. The migration rate was lower in cells treated with 100 nM AZD1152-HQPA than that in untreated cells. OS, osteosarcoma; HQPA, hydroxyquinazoline-pyrazol-anilide.

In order to investigate the effect of Aurora-B inhibition on U2-OS cell growth, AZD1152-HQPA, a specific inhibitor of Aurora-B, was used to suppress Aurora-B expression in the U2-OS cells. The cells were treated with various concentrations (0, 5, 10, 50, 100 and 500 nM) of AZD1152-HQPA, and MTT assays were performed to measure the inhibitory effect of AZD1152-HQPA on U2-OS cells proliferation. The results of the MTT assays revealed that AZD1152-HQPA inhibited U2-OS cell proliferation in a dose- and time-dependent manner (Fig. 2A). The IC50 value was 146 nM for 24 h. In the colony formation assays, the results showed that the colony formation rate in the cells treated with 100 nM AZD1152-HQPA was lower than in that in the untreated cells (Fig. 2B). Furthermore, western blot analysis showed that Aurora-B protein expression was downregulated by AZD1152-HQPA in a dose-dependent manner (Fig. 3A). These results showed that Aurora-B inhibition was capable of suppressing U2-OS cell growth *in vitro*, which suggested that Aurora-B may be a promising target for the treatment of OS.

### Inhibition of Aurora-B suppresses U2-OS cell migration and invasion in vitro

According to the IC50 value, the appropriate concentration of AZD1152-HQPA for wound healing migration and Transwell invasion assays was determined. To examine the effect of Aurora-B inhibition on the mobility of U2-OS cells, the migration and invasion were measured using wound-healing and Transwell assays, respectively. The cells were treated with 100 nM AZD1152-HQPA for 24 h. In the Transwell invasion assays, the invasion of the cells treated with AZD1152-HQPA was significantly inhibited when compared with that of the untreated cells (75.6±7.4 and 214.5±22.4 cells/high power field, respectively, P<0.05; Fig. 3B). In the wound healing assay, the results showed that the migration rate of the cells treated with AZD1152-HQPA was significantly lower than that of the untreated cells (23.7±5.1 and 75.6±15.3%, respectively, P<0.05; Fig. 3C). These results suggested that Aurora-B inhibition was capable of suppressing U2-OS cell invasion and migration *in vitro.*

## Discussion

Aurora kinases are serine/threonine kinases that are essential for cell cycle control and mitosis. Mammals have three Aurora kinase family members (A, B and C), and these kinases are expressed at maximum levels during mitosis. Aurora-B, part of the CPC, is located on the chromosome arms during prophase and at the centromeres during prometaphase and metaphase. The kinase subsequently localizes to the midbody during cytokinesis. Aurora-B has been shown to be overexpressed in a number of types of cancer (11,15–17) In the present study, the expression levels of Aurora-B protein in OS tissues were examined using IHC, which revealed that the Aurora-B protein was expressed in the nucleus, and that the positive expression rate was 53.3%. Notably, the expression levels of Aurora-B protein in the OS tissues with pulmonary metastases were significantly higher than in those without distant metastases. It was indicated that Aurora-B may be involved in the development, progression and metastasis of OS, and may be a potential novel diagnostic and therapeutic target for OS.

Recent studies revealed that Aurora-B inhibition was capable of blocking cell proliferation and inducing cell apoptosis in several types of tumor (18,19). These observations have led to an interest in Aurora-B as a molecular target for cancer treatment. A number of small molecular inhibitors of Aurora-B have been developed as promising anti-tumor treatments (6,20–23). AZD1152 is a selective inhibitor of Aurora kinase activity with specificity for Aurora-B kinase (24,25). AZD1152 is a prodrug that is rapidly converted to the active moiety, AZD1152-HQPA, in plasma. AZD1152-HQPA, as a specific inhibitor of the enzymatic activity of Aurora-B, has been used for *in vitro* investigations. Preliminary studies showed that AZD1152 was active against several types of solid tumors, including colon, breast and lung cancers (13,26). However, the effect of Aurora-B inhibition in OS malignancies has yet to be fully elucidated. In the present study, which explored the effect of Aurora-B inhibition on OS cell proliferation, AZD1152-HQPA was used to inhibit Aurora-B expression in U2-OS cells. Western blot analysis revealed that Aurora-B protein expression was decreased in cells treated with AZD1152-HQPA, compared with that in untreated cells. The results of the MTT assays showed that cell proliferation was inhibited by AZD1152-HQPA in a dose- and time-dependent manner. Furthermore, in the colony formation assays, the results revealed that the colony formation rate was significantly lower in cells treated with 100 nM AZD1152-HQPA than that in untreated cells. These results indicated that the inhibition of Aurora-B was capable of suppressing U2-OS cell growth *in vitro.*

Notably, studies recently showed that the upregulated expression of Aurora-B was associated with tumor cell metastasis, and that the downregulation of Aurora-B was capable of inhibiting cell invasion and migration in various types of tumors (11,14,27,28). In the present study, which investigated the effect of Aurora-B inhibition on OS cells, U2-OS cells were treated with 100 nM AZD1152-HQPA, and the migration and invasion of the U2-OS cells were measured using wound healing and Transwell invasion assays, respectively. The results showed that the migration rate and cell invasion were significant lower in cells treated with AZD1152-HQPA than in untreated cells. This suggested that the downregulation of Aurora-B was capable of inhibiting U2-OS cell invasion and migration *in vitro*.

In conclusion, this study indicated that Aurora-B may be involved in the development, progression and metastasis of OS, and that targeting Aurora-B may be a potential treatment strategy for OS management. However, in the present study the number of OS tissues was low. Furthermore, the tumor microenvironment is important in tumor development, progression and metastasis and therefore, further experiments *in vivo* are required to elucidate the potential of Aurora-B as a target for the treatment of OS metastases and a predictor of prognosis.
